# Clinical outcomes of dengue virus infection in pregnant and non-pregnant women of reproductive age: a retrospective cohort study from 2016 to 2019 in Paraná, Brazil

**DOI:** 10.1186/s12879-021-06985-w

**Published:** 2022-01-04

**Authors:** Beatris Mario Martin, Allan Arnold Evans, Denise Siqueira de Carvalho, Silvia Emiko Shimakura

**Affiliations:** Federal University of Paraná, Curitiba, Brazil

**Keywords:** Dengue, Severe dengue, Pregnancy, Maternal mortality

## Abstract

**Background:**

The increasing number of dengue cases worldwide implies a greater exposure of at-risk groups, such as pregnant women. DENV infection during pregnancy has been increasingly associated with unfavorable outcomes, but the evolution of the disease and its clinical outcomes remain unclear. The objective of this study was to characterize dengue cases in reproductive aged women by comparing the development of the disease in pregnant and non-pregnant women.

**Methods:**

A population based retrospective cohort study that used data reported in the Brazilian Mandatory Notifiable Diseases Information System from 2016 to 2019 in Paraná, Brazil. We compared sociodemographic, clinical, and laboratory variables between pregnant and non-pregnant women. Hospitalization and disease severity classification (Dengue, Dengue with warning signs, Severe Dengue) were considered outcome variables.

**Results:**

The two groups had differences in the year of notification, age distribution, and region of residence. Laboratory investigation was more frequent among pregnant women, and DENV-2 prevailed in both groups. The risks of hospitalization and development of Severe Dengue were higher in pregnant women. There were no deaths observed among pregnant women.

**Conclusion:**

This study identified pregnancy as a risk factor for an increase in the severity of DENV infection. It reinforces the importance of identifying early signs of complication, close monitoring, and adequate treatment for pregnant women.

## Background

The number of dengue cases has increased eightfold in the last 20 years worldwide. It is estimated that 3.9 billion people live in areas with a risk of dengue transmission [1]. In Brazil, approximately 1.5 million dengue cases were reported in 2019 [2], representing a 5.8-fold increase in comparison with the previous year [3]. Despite having the lowest incidence of cases (165.2 cases per 100,000 population in 2019) in comparison to other regions of the country, Southern Brazil also registered an increase in the number of reported dengue cases: the state of Paraná registered approximately 19,000 cases in 2018 and 113,000 in 2019. This increase in cases could possibly be related to the reintroduction of the dengue virus serotype 2 (DENV-2) during this period [4–7]. Such an expressive increase can directly result in a greater exposure of at-risk groups, such as pregnant women, in addition to developing severe forms of the disease.

DENV infection is a febrile illness that can range from asymptomatic cases to death [8]. In Brazil, people presenting a fever associated with at least two of the following symptoms such as: nausea, vomiting, rash, myalgia, headache, retroorbital pain, petechiae, positive tourniquet test or leukopenia, and with a relevant epidemiological exposure (that lives or had been traveling to an endemic area in the last 14 days) should be investigated for DENV infection [9]. During the initial assessment, the clinician classifies a suspected case of dengue into one out of three levels of severity: Dengue, Dengue with warning signs (abdominal pain, persistent vomiting, mucosal bleeding, lethargy, liver enlargement or an increase in hematocrit concurrent with a rapid decrease in the platelet count), and Severe dengue (severe plasma leakage, severe bleeding, or severe organ involvement). The health services refer in-hospital management for all pregnant women, regardless of dengue severity [8]. In such cases, diagnostic laboratory tests are also prioritized [8, 9]. The reason for such conduct refers to suggesting that this disease is associated with severe clinical conditions in pregnant women, with an eightfold increase in the risk of death [10] and a high incidence of thrombocytopenia, hemorrhagic manifestations, premature delivery, miscarriage, and stillbirth [11, 12].

Most studies investigating the relationship between dengue and pregnancy rely on data from referral health services. However, this may cause a selection bias because these studies probably include more severe cases [13]. Therefore, the objective of this study is to characterize dengue cases in women of reproductive age that were registered in Paraná State, Brazil, from January 2016 to December 2019 by comparing the development of the disease in pregnant and non-pregnant women.

## Methods

This is a population based retrospective cohort study of pregnant and non-pregnant women of reproductive age diagnosed with dengue based on data available in the Brazilian Mandatory Notifiable Diseases Information System (SINAN) from January 2016 to December 2019.

### Data source

In Brazil, health professionals must report all febrile individuals with typical dengue clinical manifestations to Epidemiological Surveillance. The protocol is to investigate all suspected cases. However, during epidemic periods, not all cases are confirmed by laboratory tests [8]. In this manuscript, we included all cases confirmed using clinical/epidemiological or laboratory criteria. Data regarding suspected cases are collected from the Dengue and Chikungunya Fever Report Form that contain clinical, laboratory, and identification data, as well as co-existing health conditions information—such as pregnancy. It also states whether or not the patient needed hospitalization and the outcome (cured or died). In the last case, whether or not the death was due to a dengue virus infection [9].

Since 2014, clinicians classify dengue cases according to disease severity as Dengue, Dengue with warning signs, and Severe Dengue, following the World Health Organization classification [14].

The diagnostic laboratory tests performed to confirm the disease are defined according to the time that has elapsed since the onset of symptoms. Children, pregnant women, the elderly, and patients with comorbidities must be investigated through laboratory tests even during epidemic periods [15].

### Data selection

In Brazil, women aged between 10 and 49 are considered in the reproductive period in accordance with the Manual of the Committees of Maternal Mortality [16]. We excluded the records classified as “under investigation” or “inconclusive” at the time of data collection. We also excluded cases reported as “discarded” from this study. Records where the pregnancy status was incomplete or classified as “ignored” were excluded as well.

### Variables

We divided the variables selected for our analysis into two sets: sociodemographic and clinical variables. The sociodemographic variables included: age*,* race*,* years of education*,* year of notification*,* and region of residence*.* Also, we considered age into four age groups: 10–19, 20–29, 30–39, and 40–49 years of age; and race into three categories: White, Black (Brown and Black), and others (Asian descendants and native Brazilians) [17]. We classified years of education as incomplete primary education, incomplete *s*econdary education, and completed *s*econdary education [18]. Finally, we defined the region of residence according to the corresponding health macroregion. Paraná State Health Secretary groups the municipalities into 22 health regions divided into four macroregions (Northwestern, Northern, Western, and Eastern). The clinical variables selected were comorbidities, characteristic signs of dengue, and laboratory diagnosis. The comorbidities considered in this study were: diabetes, hypertension, autoimmune diseases, acid peptic disease, chronic kidney disease, hematological diseases, and liver diseases. The characteristic clinical signs of dengue were fever, myalgia, headache, rash, vomiting, nausea, back pain, conjunctivitis, arthritis, severe arthralgia, petechiae, leukopenia, positive tourniquet test, and retro-orbital pain. The laboratory tests available for investigation from the database included: immunoglobulin (Ig)M detection, detection of viral antigen nonstructural protein 1 (NS1), virus isolation (culture), reverse-transcriptase polymerase chain reaction assay (RT-PCR), histopathology, and immunohistochemical staining. A case investigated by at least one of these tests is considered a laboratory investigated case, and if any test confirmed dengue infection, it is a laboratory confirmed case.

### Exposure and outcomes

The principal exposure factor of this study was the presence or absence of pregnancy. As main outcomes, we considered the level of severity classification (Dengue, Dengue with warning signs, Severe Dengue), and hospitalization*.*

### Statistical analysis

Bivariate analysis included evaluation of pregnancy status stratified by each sociodemographic, clinical and laboratory variable. Wald chi-square statistics were calculated to assess significant differences between pregnant and non-pregnant women by each variable separately. Estimated odds ratios, confidence intervals, and *P* values were calculated using EPI INFO 7.2.4.0® software (Centers for Disease Control and Prevention, Atlanta, GA, USA). *P* values less than 0.20 were used to select the variables included in the multivariable analysis.

Multivariable analysis included unconditional logistic regressions on two outcomes: severity level classification and hospitalization. A *P* value less than 0.05 was used as an indicator that a difference between groups may exist.

### Ethics

This research project was approved by the ethics committee of the Federal University of Paraná (CAAE 62213916.1.0000.0102).

## Results

**Fig. 1:**
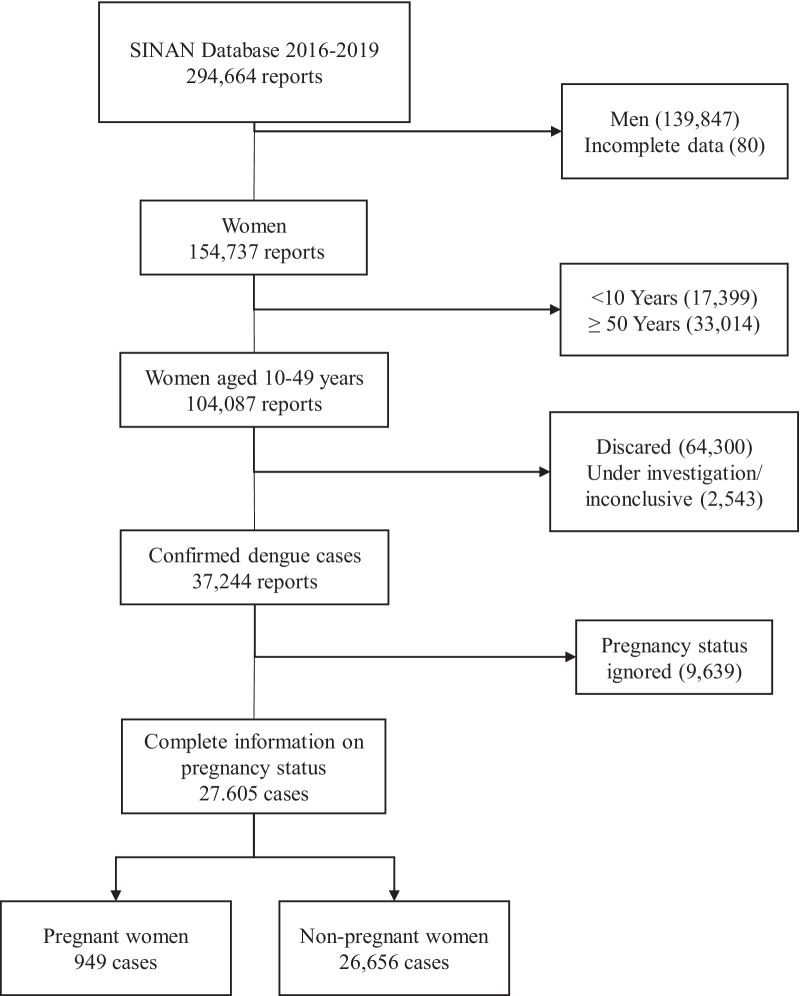
Flowchart of case selection

In the evaluated period, we found a total of 294,664 reports, among which 154,737 were women (52.5%), and 104,087 of them were of reproductive age (35.3%). Out of the total number of women from the reproductive age group (104,087), 37,244 (35.8%) were confirmed as dengue cases classified by the clinical-epidemiological or laboratory criteria. Of these 37,244 cases, 27,605 (74.1%) had complete information on their pregnancy status. We included in our analysis these 27,605 women, in which 949 (3.44%) of them were confirmed pregnant (Fig. 1).

Among the cases included in the analysis, 13,708 (49.7%) were reported in 2016 and 13,285 (48.1%) in 2019. Of those, 20,082 (72.3%) were classified as white women, 15,426 (55.9%) with the completion of secondary education. In 7723 (28.0%) cases, the information pertaining to years of education was missing. The distribution of cases according to region of residence was distributed in the Northwest, 8825 (32.0%), the West, 8197 (29.7%), the North, 7694 (27.9%), and the East 2889 (10.5%) cases.

The comparison between pregnant and non-pregnant women found no significant differences in years of education and race. The year 2016 corresponds to the highest number of pregnant women reported, 502 cases (52.9%) of all dengue cases among the pregnant women group. The distribution of dengue cases according to the year of notification and pregnancy status suggests a smaller proportion of dengue among pregnant women in 2019 relative to 2016 (Table 1). We also found significant differences between pregnant and non-pregnant women in the age and region of residence distributions (Table 1).

**Table 1:** Characteristics of the reproductive aged women registered with dengue fever according to their pregnancy status

	Pregnant women N = 949 (%)	Non-pregnant women N = 26,656 (%)	OR [CI 95%]	*p*-value
Year of notification				
2016	502 (52.9)	13,206 (49.5)	REF^k^	
2017	10 (1.1)	310 (1.2)	0.85 [0.45–1.60]	0.6129
2018	12 (1.2)	280 (1.1)	1.13 [0.63–2.02]	0.6873
2019	425 (44.8)	12,860 (48.2)	0.87 [0.76–0.99]	**0.0369**
Race^a^				
White	689 (72.6)	19,393 (72.8)	REF	
Black	216 (22.8)	6091 (22.9)	1.00 [0.85–1.17]	0.9813
Others	8 (0.9)	298 (1.1)	0.76 [0.37–1.53]	0.4375
Education^b^				
Incomplete P. E.^c^	29 (3.1)	803 (3.0)	REF	
Incomplete S. E.^d^	122 (12.9)	3502 (13.1)	0.97 [0.64–1.46]	0.8640
Complete S. E.^e^	553 (58.3)	14,873 (55.8)	1.03 [0.70–1.51]	0.8807
Age (years)				
10–19	152 (16.0)	5266 (19.8)	REF	
20–29	461 (48.6)	7120 (26.7)	2.24 [1.86–2.70]	**0.0000**
30–39 years	256 (27.0)	7385 (27.7)	1.20 [0.98–1.47]	**0.0782**
40–49 years	80 (8.4)	6885 (25.8)	0.40 [0.31–0.53]	**0.0000**
Macroregions				
Eastern	150 (15.8)	2739 (10.3)		
Western	269 (28.4)	7928 (29.7)	0.71 [0.58–0.86]	**0.0007**
Northern	244 (25.7)	8581 (32.2)	0.52 [0.42–0.64]	**0.0000**
Northeastern	286 (30.1)	7408 (27.8)	0.62 [0.51–0.76]	**0.0000**
Comorbidities				
Presence of at least one comorbidity? (Y/N)	50 (5.3)	1278 (4.8)	1.10 [0.83–1.48]	﻿0.5024
Diabetes	16 (1.7)	297 (1.1)	1.52 [0.92–2.53]	**0.1046**
Hematological diseases^f^	6 (0.6)	111 (0.4)	1.52 [0.67–3.47]	﻿0.3181
Liver diseases^g^	2 (0.2)	119 (0.5)	0.47 [0.12–1.91]	﻿0.2916
Chronic kidney disease	3 (0.3)	90 (0.3)	0.94 [0.30–2.97]	﻿0.9131
Hypertension	25 (2.6)	804 (3.0)	0.87 [0.58–1.30]	﻿0.4987
Acid-peptic diseases	3 (0.3)	120 (0.5)	0.70 [0.22–2.21]	﻿0.5457
Autoimmune diseases^h^	4 (0.4)	121 (0.5)	0.93 [0.34–2.52]	﻿0.8838
Laboratory diagnosis test				
Submit to at least one diagnostic test? (Y/N)	726 (82.6)	16,067 (66.7)	2.37 [1.99–2.83]	**0.0000**
Serotype identification^i^				
DENV-1	71 (42.3)	1058 (43.2)	REF	
DENV-2	80 (47.6)	1260 (51.4)	0.95 [0.68–1.32]	0.7421
DENV-3	0 (0.0)	2 (0.1)	0.00	–^k^
DENV-4	17 (10.1)	132 (5.4)	1.92 [1.10–3.36]	**0.0223**
Characteristic signs and symptoms				
Fever	513 (54.1)	16,900 (63.4)	0.68 [0.60–0.77]	**0.0000**
Myalgia	523 (55.1)	16,771 (62.9)	0.72 [0.64–0.82]	**0.0000**
Headache	540 (56.90	17,208 (64.6)	0.73 [0.64–0.83]	**0.0000**
Exanthema	212 (22.3)	6086 (22.8)	0.97 [0.83–1.14]	0.7244
Vomiting	177 (18.7)	4350 (16.3)	1.18 [1.00–1.39]	**﻿0.0568**
Nausea	288 (30.4)	9081 (34.1)	0.84 [0.73–0.97]	**0.0175**
Back pain	247 (26.0)	8427 (31.6)	0.76 [0.66–0.88]	**0.0003**
Conjunctivitis	14 (1.5)	812 (3.0)	0.48 [0.28–0.81]	**0.0064**
Arthritis	87 (9.2)	2895 (10.9)	0.83 [0.66–1.04]	**﻿0.0993**
Severe arthralgia	15 (15.8)	5034 (18.9)	0.81 [0.68–0.96]	**0.0173**
Petechiae	82 (8.6)	2522 (9.5)	0.91 [0.72–1.14]	0.3956
Leukopenia	52 (5.5)	1704 (6.4)	0.85 [0.64–1.13]	0.2581
Tourniquet test	48 (5.1)	1859 (7.0)	0.71 [0.53–0.95]	**0.0228**
Retro-orbital pain	294 (31.0)	9633 (36.1)	0.79 [0.69–0.91]	**0.0012**
Level of severity classification				
Dengue	928 (97.8)	26,314 (98.7)	REF	
Dengue with warning signs	17 (1.8)	321 (1.2)	1.51 [0.92–2.46]	**﻿0.1027**
Severe dengue	4 (0.4)	21 (0.1)	5.40 [1.85–15.77]	**0.0020**
Hospitalization^j^				
Hospitalized? (Y/N)	107 (13.9)	1100 (5.2)	2.93 [2.37–3.63]	**0.0000**
Outcome				
Death	0	12 (0.1)	0.00	–^m^

Bold indicates that the *p*-value less than 0.20

^a^Data was missing in 874 (3.3%) notifications

^b^Data was missing in 7723 (28.0%) notifications

^c^Incomplete primary education

^d^Incomplete secondary education

^e^Complete secondary education

^f^Anemia, macrocytosis, hemolytic anemia, leukemia, lymphoma, trombocytopenia

^g^Hepatitis, fatty liver disease, hepatobiliary disorders, etc.

^h^Lupus erythematosus, rheumatoid arthritis, multiple sclerosis, chronic autoimmune thyroiditis, etc.

^i^Serotype was identified in 168 pregnant women and in 2452 non-pregnant women

^j^Information about hospitalizations was available in 21,850 (79.2%) notifications

^k^REF: variable used as reference for calculating OR

^l^There were no cases of DENV-3 identified among pregnant women

^m^There were no deaths among pregnant women

Hypertension and diabetes mellitus were the most frequent comorbidities, accounting for 829 (3.0%) and 313 (1.1%) respectively of the total cases. There was no significant difference in the proportion of comorbidities between pregnant and non-pregnant women (Table 1). The data reporting characteristic dengue signs and symptoms were missing in 7119 (25.8%) cases, all from 2016. Among the complete data, we observed fewer reports of fever, myalgia, headache, nausea, back pain, conjunctivitis, articular pain, positive tourniquet test and retro orbital pain in pregnant women (Table 1).

Among all cases included in the analysis 16,793 (60.8%) had performed at least one diagnostic test; the test results were positive in 16,286 cases (97.0%) and those cases were considered as laboratory confirmed cases*.* Regarding the women tested (16,793), 2620 (16.6%) had the serotype test, and DENV-2 was identified in 1340 (51.1%) of them. Pregnant women were tested more frequently (82.6%) than non-pregnant women (66.7%) (OR 2.37 [1.99–2.83]). Among the pregnant women who had the DENV serotype identified (116), DENV-2 was found in 80 (47.6%) cases, indicating a predominance of this serotype. Serotype 4 (DENV-4) was more frequent in pregnant women than in non-pregnant women (OR 1.92 [1.10–3.36]) (Table 1).

Information about hospitalizations was available in 21,850 (79.2%) notifications. Among those where the hospitalization information was complete, the risk of hospitalization in pregnant women was higher than in the non-pregnant women group (OR 2.93 [2.37–3.63]), as well as the risk of developing Severe Dengue (OR 5.40 [1.85–15.77]) (Table 1).

Regarding the laboratory confirmed cases (16,286), 707 were pregnant women and 15,576 were non-pregnant women. Among the pregnant women that were confirmed with a laboratory diagnosis, 95 (15.3%) were hospitalized, 14 (2.0%) presented Dengue with warning signs, and 4 (0.4%) Severe Dengue. Among the non-pregnant women that were confirmed with a laboratory diagnosis, 906 (6.8%) were hospitalized, 274 (1.8%) presented Dengue with Warning Signs, and 19 (0.1%) Severe Dengue. We performed a sensitive analysis using only the laboratory confirmed cases, the risk of hospitalization (OR 2.49 [1.98–3.13]) and the development of Severe Dengue (OR 4.67 [1.59–13.77), which were still present in pregnant women.

The variable year of notification, age, region of residence, and presence of diabetes were selected in the bivariate analysis, considering a significance level of 0.20. The impact of these variables on each outcome (hospitalization and Severe Dengue) were evaluated by unconditional logistic regression (Tables 2 and 3).

**Table 2:** Logistic regression showing unadjusted and adjusted odds ratio for hospitalization in women of reproductive age

	Unadjusted		Adjusted	
	OR [CI 95%]	*p*-value	OR [CI 95]	*p*-value
Pregnancy status				
Pregnancy (yes/no)	**2.93 [2.37–3.63]**	**0.0000**	﻿2.64 [2.04–3.43]	**0.0000**
Year of notification				
2016	REF^a^			
2019	1.05 [0.93–1.18]	0.4238		
Age (years)				
10–19	REF		REF	
20–29	1.27 [1.06–1.52]	**0.0085**	1.22 [0.99–1.51]	0.0673
30–39	1.32 [1.10–1.57]	**0.0025**	﻿1.39 [1.13–1.71]	**0.0017**
40–49 years	1.19 [0.99–1.43]	﻿0.0688	﻿1.27 [1.02–1.57]	**0.0312**
Region of residence				
Eastern	REF		REF	
Western	0.48 [0.38–0.59]	**0.0000**	﻿0.42 [0.30–0.58]	**0.0000**
Northern	0.31 [0.25–0.39]	**0.0000**	﻿0.29 [0.21–0.41]	**0.0000**
Northeastern	0.32 [0.25–0.40]	**0.0000**	﻿0.28 [0.20–0.39]	**0.0000**
Comorbidities				
Diabetes (yes/no)	1.61 [1.05–2.49]	**0.0283**	﻿1.59 [1.02–2.46]	**0.0388**

Bold indicates that the *p*-value less than 0.05

Data of hospitalization was available in 20,643 non-hospitalized cases and 1207 hospitalized cases

^a^REF: variable used as reference for calculating OR

**Table 3:** Logistic regression showing unadjusted and adjusted odds ratio for severe dengue in women of reproductive age

	Unadjusted		Adjusted	
	OR [CI 95%]	*p*-value	OR [CI 95%]	*p*-value
Pregnancy status				
Pregnancy (yes/no)	﻿5.40 [1.85–15.77]	**0.0020**	﻿5.47 [1.86–16.03]	**0.0020**
Year of notification				
2016	REF^a^		REF	
2019	0.34 [0.14–0.87]	**0.0235**	﻿0.35 [0.14–0.88]	**0.0259**
Age (years)				
10–19	REF			
20–29	1.25 [0.37–4.27]	0.7213		
30–39	1.60 [0.49–5.19]	0.4361		
40–49	0.97 [0.26–3.62]	0.9668		
Region of residence				
Eastern	REF			
Western	1.59 [0.34–7.35]	0.5550		
Northern	0.49 [0.08–2.94]	0.4367		
Northeastern	2.07 [0.46–9.33]	0.3451		
Comorbidities				
Diabetes	3.59 [0.48–26.97]	0.2537		

Bold indicates that the *p*-value less than 0.05

^a^REF: variable used as reference for calculating OR

In the multivariable analysis, pregnancy, age, place of residence, and presence of diabetes were significant in hospitalized cases. Pregnancy and diabetes increased the risk of hospitalization. We found for all age groups, compared with 10 to 19 years old, increased the risks of hospitalization. For all macroregions compared with the Eastern macroregion there was a decrease in hospitalization risks. The year of notification did not show a significant difference (Table 2). The development of Severe Dengue was higher in pregnant women and lower in the year 2019 in the multivariable model analysis (Table 3).

## Discussion

In this study, among all the cases of dengue in the reproductive aged women pregnancy was associated with a higher risk of hospitalization and Severe Dengue. This risk was still present when analyzing only the laboratory confirmed cases, and in the multivariable model.

A population based retrospective cohort study allows the inclusion of patients with a wide spectrum of severity because the records are not restricted to severe cases which have resulted in hospitalization. Thus, in turn, more accurately reflect in the behavior of the disease. The SINAN database used as a data source is frequently used in scientific research and the quality of the information obtained is related to accuracy in the completion of the report form. Information regarding identification, clinical and laboratory data is usually better answered [19]. In the period evaluated, there were differences in the completeness of the report form. Missing data on the pregnancy status although mainly detected in 2016, comprised 25.9% of all registered dengue cases in reproductive aged women. We had to exclude these records from the analysis. In Paraná, studies conducted before 2016 indicated that almost all dengue report forms are investigated within 7 days [20] and have a 90% or higher completion rate with gestation data [21]. The occurrence of an epidemic period and the high number of notifications may contribute to a decrease in data quality. Variables such as years of education, race, and occupation are typically poorly completed in report forms of notifiable diseases [19]. An evaluation regarding how complete the information regarding the years of education was with the dengue cases reported to SINAN from the Northeastern and Southeastern regions in Brazil showed that this information was correctly answered in less than 30% of the cases in at least 8 of the 13 evaluated capital cities [22]. We found a 70% rate of data completion relating to years of education. Also, the data found in this study on racial distribution and years of education agree with the profile of Paraná’s population: 65.5% are white [23], and 77.4% of the population have more than 8 years of education [24].

The definition for dengue cases in Brazil consists of clinical-epidemiological and laboratory criteria [8], and we decided to include both criteria in our analysis. The use of clinical criteria, or diagnostic models, has the advantage of allowing for an early recognition of suspected cases. Although, it can impair ranking in severity levels and correct diagnosis [25]. The analysis of the laboratory confirmed cases had similar findings when compared with the analysis of all cases, suggesting that the clinical-epidemiological criteria was reliable. Nevertheless, there were differences in the proportions of laboratory investigations and diagnosis in pregnant and non-pregnant women. It is not an unexpected finding considering the orientation from the Brazilian Health Department prioritizing laboratory tests in pregnant women [8]. A national cross-sectional study [26] that evaluated pregnant women with dengue from 2007 to 2015 found that 81.6% of the cases were investigated using laboratory tests. These data results are similar to those found in our study, where 82.6% of pregnant women were tested.

The gestation period is characterized by several anatomical and physiological alterations in the female body, such as hypervolemia, tachycardia, increased capillary permeability, hemodilution, leukocytosis, and thrombocytopenia [27]. The role of these modifications during DENV infection are not clear. However, their presence may complicate both the assessment of the clinical picture of dengue and the identification of warning and severity signs and symptoms, overlapping or even compensating pregnancy physiological changes (gestation causes leukocytosis and dengue leukopenia) [28]. The missing data describing signs and symptoms characteristics of dengue infection represents 25.8% of all cases, thus, limiting the analysis of our findings. Nevertheless, warning and severity signs and symptoms completeness was higher. The comparison of pregnant and non-pregnant women found no differences, except for a decrease in the platelet count which was less frequently described in pregnant women. A hospital based study aiming to evaluate hematological alterations in patients with DENV infection found that most pregnant women presented an uncomplicated form of the disease, and even though 57% of the pregnant women presented thrombocytopenia, this alteration was less frequent than in the general population (82%) [29]. Those findings are in accordance with ours, however the differences in both studies’ designs should be considered when analyzing this result. Our study used data from National Surveillance, and as previously exposed, the quality of data relies on the completeness of the report form. Since a low platelet count is expected during pregnancy [27] and is also frequently associated with dengue infection during pregnancy [12], this asks a question with reference to the quality of the data. Despite this result, pregnancy was associated with Severe Dengue.

The year of notification was also associated with Severe Dengue, when comparing the two epidemic years—2016 and 2019. Dengue epidemics are associated with *Aedes aegypti* circulation, DENV serotypes and number of susceptible populations. Globalization, urbanization, and poverty are factors that contribute to mosquito dissemination and reduction of the period between dengue epidemics [30, 31]. From 1990 to 2015, it is possible to observe an intensification of dengue epidemics—almost 50% of all epidemics registered happened in the last 5 years [32, 33]. In Brazil, the years 2015/2016 and 2019 were markedly epidemic years [34]. During 2019, the predominant serotype was DENV-2, which has been linked in previous publications to more severe cases of the disease, in addition to higher hospitalization rates—especially among children and adolescents [35–37]. In this study, the multivariable analysis associated the year of 2016 with a higher risk of severe dengue and more frequent than 2019. The year 2016 represents a larger proportion of cases in this population, and during 2017 and 2018 we observed a reduction of cases. Although variations in dengue incidence are expected, this drop in cases raises some questions, such as a possible influence of the Zika virus (ZIKV) epidemic that took place in Brazil and Latin America in the year of 2016. This epidemic was followed by a dramatic decrease of dengue and Zika cases in 2017 and 2018 [38, 39]. Several hypotheses were considered as possible explanations, such as changes in epidemiological surveillance systems, cross-immunity and impact of climate change and multiple arboviruses in mosquito population [38]. In a previous debate, the protective cross-immunity generated by the simultaneous circulation of more than one type of arboviruses was considered as the most probable explanation by a panel of experts [38]. Additionally, a study conducted in two different Brazilian states found that between 2000 and 2019 population susceptibility to dengue infection had reduced related to previous dengue and Zika infection, resulting in cross-immunity which could partially explain the decrease in dengue cases observed in 2017 and 2018 [39]. In 2019, the increase in the number of cases may be related to the DENV-2, which has been circulating in Brazil since 2015, but initially affected the Northeastern region of the country [40]. In Paraná, this serotype has been detected since 2016 in a few cases, but, until 2018, DENV-1 was the predominant serotype [7]. The association of severe dengue in 2016 may be a bias caused by the higher number of cases in this year. In the evaluated population, the proportion of DENV-4 in pregnant women was higher than in non-pregnant women.

Hospitalizations were related to pregnancy, but also by the presence of diabetes, age, and place of residence. All regions of residence compared with the Eastern macroregion had a decreased risk of hospitalization. This lower hospitalization could not be explained by serotype, since in both epidemic years, this macroregion was affected by the same dominant serotype as the others. In the Western macroregion, DENV-4 is present for a long time [7] but with no impact in severity or hospitalization. The Eastern macroregion had the smallest number of dengue cases in Paraná State, and this may reflect in hospitalization rates, since health professionals and services are less experienced in attending dengue cases [41]. Hospitalization is encouraged by Brazilin Health department in dengue cases in pregnant women or people with comorbidities [8], because of previous association with increased risk of severity in dengue infections [9, 42]. This association was observed in a Mexican cohort evaluating risk factors associated with higher hospitalization and mortality. It found that pregnancy, age (younger than ten and older than 60 years), diabetes, hypertension, chronic kidney disease were risk factors for both outcomes [43]. Also, a higher mortality was described in a study that compared data from the Brazilian dengue, live-birth, and mortality information systems between 2007 and 2012. Based on this, the authors assessed the impact of dengue infection on maternal mortality and found an eightfold increase in the risk of death in pregnant women diagnosed with dengue, a 27-fold increase in cases with laboratory confirmation, and a 451-fold increase when the final classification corresponded to the most severe form of the disease [10]. Nevertheless, the observation that both pregnancy and diabetes were associated with higher hospitalizations may be related with the quality of health care. This possibility is reinforced by the fact that pregnancy was associated with Severe Dengue and hospitalization, but no cases resulted in death.

Although this study presents evidence associating pregnancy and Severe Dengue, there are some limitations that should be considered in the analysis. First, this population based retrospective cohort study was conducted using secondary data obtained from the SINAN database, and the quality of the information is related with the completeness of the report filings. As we have already discussed, some variables were partially incomplete. Second, we used only the data available in SINAN to evaluate mortality and pregnancy, and those data could be improved, if SINAN was linked with the Mortality Information System (SIM) and Live Birth Information System (SINASC). Improving the completeness of SINAN data and the linkage of several Brazilian Information Systems could ameliorate the knowledge pertaining to the impact of pregnancy in dengue infection.

## Conclusion

By demonstrating the association of pregnancy with Severe Dengue and hospitalization in a broad population, this study contributes to understanding the evolution of dengue during the gestation period. The role of severity ranking, risk factor identification and the early management of dengue infection may result in the possibility of preventing the evolution to Severe Dengue and death. Thus, the identification of pregnancy as a risk factor for unfavorable clinical outcomes reinforce the support for healthcare professionals and services and reflect the importance of identifying early signs of bleeding, and facilitate hospitalization, monitoring, and adequate treatment for pregnant women.

## Data Availability

The datasets used and/or analyzed during the current study are available from the corresponding author on reasonable request.

## Abbreviations

DENV: Dengue virus
HELLP: Hemolysis, elevated liver enzymes, low platelet count
SIM: Mortality Information System (in portuguese: *Sistema de Informação de Mortalidade*)
SINAN: Brazilian Notifiable Diseases Information System (in portuguese: *Sistema de Informações de Agravos de Notificação Obrigatória*)
SINASC: Live Birth Information System (in portuguese: *Sistema de Informação sobre Nascidos Vivos*)
ZIKV: Zika virus
